# P05.08. A study of complementary therapies and counselling: an integrative model for refugee health care

**DOI:** 10.1186/1472-6882-12-S1-P368

**Published:** 2012-06-12

**Authors:** J Singer, J Adams

**Affiliations:** 1 University Centre for Rural Health, Sydney University, Lismore, NSW, Australia; 2 Victorian Foundation for Survivors of Torture, Melbourne, Australia

## Purpose

Two qualitative research studies were conducted to examine the experiences of refugee women involved in a complementary therapies (CT) program and to investigate counsellors’ reasons for referral to CT. Combined, the two studies aim to provide a comprehensive overview of this innovative model of integrative health care. The Victorian Foundation for Survivors of Torture, known as ‘Foundation House’, is a mental health service for refugees and asylum seekers in Melbourne, Australia. The organisation was established in 1987 and within two years incorporated a CT program with the aim to provide a holistic and culturally relevant health care approach. The inclusion of CT (naturopathy, Western herbal medicine, massage, yoga, nutritional and dietary advice) as a core component of the service delivery positioned the organisation as one of the first Western-based torture trauma services to fully integrate psychological care and non-biomedical health care practices.

## Methods

In-depth interviews with 12 current clients engaged in the CT program examined their experiences of the CT program. A focus group with 10 counsellors who had experience in referring clients to the CT program was also conducted to investigate their reasons for referral and understanding of CT.

## Results

Study findings are framed under three ‘modes of action’: relationship, cultural familiarity and somatic presentations. In the integrative model at Foundation House, counsellors’ understandings of how CT work and their reasons for referral correlate with the clients’ experiences of CT. Of significance, are the ways in which these parallel experiences and beliefs intersect and inform understandings about the role of CTs at Foundation House, and in turn, the broader refugee health care context.

## Conclusion

Our combined findings extend current notions of holistic refugee health care, and indeed integrative health care, to include a model in which psychological and complementary therapies are practiced within a collaborative model of care.

